# Correlations of external social capital in social organizations providing integrated eldercare services with medical care in China

**DOI:** 10.1186/s12913-022-07508-2

**Published:** 2022-01-25

**Authors:** Ling Tang, Zhongliang Bai, Kai Ji, Ying Zhu, Ren Chen

**Affiliations:** 1 School of Health Services Management, Anhui Medical University, Hefei, 230032 China; 2 Suzhou Hospital Affiliated to Anhui Medical University, Suzhou, 234099 China

**Keywords:** External social capital, Integrated eldercare services with medical care, Social organizations

## Abstract

**Background:**

This study aimed to explore the external social capital of social organizations (SOs) providing integrated eldercare services with medical care in Anhui Province, China. Specifically, we studied the current situation and influencing factors of external social capital and its six dimensions.

**Methods:**

We conducted a cross-sectional study in Anhui Province, China using a multi-stage stratified random sampling method. We employed Pearson correlation analysis and a binary logistic regression model.

**Results:**

The final analysis included 49 SOs. Most organizations had a high score in norm dimension (81.6%), participation (61.2%), trust (65.3%), common language (65.3%), and social capital (63.3%). After adjusting for all covariate variables, integrated eldercare services with medical care SOs which served more than 65 elderly people were likely to report lower score in social capital.

**Conclusions:**

By examining the current situation of integrated eldercare services with medical care SOs in China, this study enriched the relevant evidence of integrated medical and nursing SOs and provides a certain reference value for relevant management departments when formulating policies.

**Supplementary Information:**

The online version contains supplementary material available at 10.1186/s12913-022-07508-2.

## Introduction

With the rapid aging of the population, numerous countries are facing great challenges [1]. By the end of 2020, China’s population aged 65 and above has reached 191 million, accounting for 13.50% of the total population [2]. As one of the fastest aging countries globally, the aging population of China is expected to reach 363 million (24.8% of the total population) by 2030 [3]. As a result, concerns have been expressed regarding how to maintain the health and well-being of the elderly. It has become a consensus that the whole society should support the elderly. Social organizations are increasingly burdened with social responsibilities, particularly in the elderly service [4, 5]. Commonly, part of the elderly who do not live at home lives in government-sponsored pension institutions. Some live in nursing homes that are operated by private investors. While the others have dwelled in SOs [6].

In contrast to traditional elderly care SOs that offer basic living services and financial support integrated medical and nursing SOs provide medical care services to elders who choose to live in institutions [7–9]. Studies have confirmed that the occurrence and development of integrated eldercare services with medical care SOs could facilitate the lives of older people to a certain extent [10]. Previous studies also show that the cultivation and enrichment of social capital contribute to the growth of SOs and the improvement of organizational ability [11, 12], to realize the mission and objectives of the organization and promote the sustainable development of SOs. Therefore, full consideration should be given to promoting the further development of these organizations.

Social capital reflects how individuals or groups can obtain resources through inter-social networks and support [13–16]. It has been suggested that social capital affects health through the following channels: promoting faster information dissemination, increasing the possibility of adopting healthy behaviors, and implementing social control over unhealthy behaviors [17]. Previous analysis showed that lifestyle factors modestly mediate the association between social capital and elderly health [18]. These studies highlight that the social capital substantially influences elderly health. As an intangible resource, social capital possesses rich connotations and elements, strong applicability, and function. Social capital is not an isolated entity [19–21]. Social support, social connection, trust, cohesion, and reciprocity all contribute in different ways to promoting coordination and cooperation of mutual benefit and improving social efficiency [22]. External social capital refers to the actual and potential resources that an organization obtains from the cooperative network established with other external stakeholders [23]. External social capital can promote SOs to better participate in the supply of pension services. However, few people pay attention to the potential of SOs to participate in China’s elderly care services.

As one of the main pension services, integrated eldercare services with medical care social organizations are critical in managing social pension system to solve the population aging problem. For instance, many older people now prefer to live in SOs [10, 24, 25]. Moreover, the government is unable to cope with the aging population and can not meet the needs of multi-level elderly care services. The combination of medical care and social organizations can provide a variety of services for the elderly, which has incomparable advantages between the government and the market [5, 24]. It is worth noting that social capital is defined and researched in terms of social networks. However, relevant knowledge regarding applying external social capital to pension social organizations remains limited, particularly the integrated eldercare services with medical care SOs.

To our knowledge, no studies to date have investigated the social capital of social organizations providing integrated eldercare services with medical care in China. In light of the Chinese aging problem, our study focused on external social capital in social organizations providing integrated eldercare services with medical care in Anhui Province, China. Consequently, from a social capital theory perspective, analyzing and evaluating the current situation and correlated factors of external social capital in SOs providing integrated eldercare services with medical care in China is critical. Eventually, we aim to provide a reference for increasing the external social capital of social organizations with integrated eldercare services with medical care.

## Materials and methods

### Study population and data collection

Based on geographical location, we recruited the subjects using a multi-stage stratified random sampling method. This sampling method ensured that a representative sample size was achieved. The study area, Anhui province of China, was stratified into three regions: South, Central, and North. Two cities were then chosen from each stratum: Anqing and Chizhou in the South, Huainan and Luan in the Middle, and Suzhou and Fuyang in the North. Following that, all districts or counties from each city were selected as sampling areas. As a result, 15 districts and counties were chosen for this study. Then, all integrated eldercare services with medical care SOs were selected from 15 districts and Counties. The questionnaire respondents are 1–3 core members of aged-related social organizations, including legal representatives, founders, leaders, and managers familiar with the organization.

Aided by local workers, skilled and trained graduate students from Anhui Medical University visited each participant and conducted face-to-face interviews. The study used structured questionnaires. The participants were verbally informed of the study’s objectives and procedures, and informed consent was obtained before the interview. We interviewed 49 social organizations, of which 49 were valid samples.

### Measure

#### Measures of external social capital

We utilized an external social capital scale to obtain social capital information. The external social capital scale is a 25-item questionnaire tool used to measure the external social capital of an organization [26, 27]. The tool consists of six dimensions: participation, trust, support, norm, common language, and common vision. In the present study [13, 26, 27], a five-point scale of Likert was adopted in the social capital questionnaire. The respondents were asked to rate their agreement, ranging from 1 (totally disagree) to 5 (totally agree). For each domain of social capital, answers to varied items were summarized to get an overall score, and a higher score indicates a better social capital status. The total score of external social capital was the sum of six dimensions. Cronbach’s α of the questionnaire was 0.907, indicating that our scale has excellent internal consistency with this population. The detailed questionnaire is shown in the supplementary file (1).

#### Measures of other variables

Information on the basic situation of SOs variables was also collected. This included region, establishment time, full-time employees, operation pattern, service content types, number and type of categories of an object (self-care, half-care, total care, and special care), number and type of age groups served, number of elderly people served, number of disabled elderly, number of fund sources, source of funds (service charges, financial subsidies, medical insurance funds, social donations, and others) and chain organization.

### Statistical analysis

Continuous variables were presented as mean ± standard deviation. Range categorical variables were presented as numbers (%). Following that, Spearman correlation analysis was performed. Then we performed a likelihood ratio test. Finally, we examined the association between social capital and influence factor using a binary logistic regression model with an enter method. Previous studies have shown that a binary logistic regression model can be used for analysis despite a smaller sample size, [28–30]. So we employed a binary logistic regression model with an enter method to investigate the relationship between social capital and possible influencing factors. We dichotomized social capital scores and each dimension of social capital into two categories by taking the average score as the cut-off [31, 32].

If the score is less than the average, it is assigned to the low group (assignment of 0), and if the score is greater than the average, it is assigned to the high group (assignment of 1). We first performed a univariate logistic regression model, followed by a logistic regression model with adjusted potential covariates (including region, establishment time, full-time employees, operation pattern, service content types, number and type of object categories, number, and type of age groups served, number of elderly people served, number of disabled elderly, number of fund sources, source of funds, and chain organization). All statistical analyses were performed using SPSS statistics software, version 26 (SPSS Inc.; Chicago, IL, USA). *P*-value ≤0.05 was considered statistically significant.

## Results

### Results of descriptive analysis

The results of SOs characteristics were shown in Table 1. This study investigated 49 integrated eldercare services with medical care SOs in six sample cities of Anhui Province. The study included 30 (61.2%) organizations from rural areas. Additionally, there were 19 (38.8%) organizations that had been established for 1–5 years. There were 22 (44.9%) organizations with more than 18 full-time employees. Only 8 (16.3%) organizations were responsible for public construction and operation. Most organizations (73.5%) provided 7–10 services. Moreover, 10 (20.4%) organizations had only one type of source of funds. The objects of organizations mainly included the following five categories: self-care, half-care, total care, particular care, and special care. A total of 26 (53.1%) and 25 (51.0%) organizations served more than 60 people and 30 disabled elderly people, respectively. A total of 45 organizations (91.8%) obtained funds through financial subsidies, and 25 (51.0%) institutions were chain organizations.

**Table 1:** Descriptive analysis results of social organizations characteristics

Variables		***N*** = 49
**Region, N (%)**	**Urban**	19 (38.8)
	**Rural**	30 (61.2)
**Establishment time, years, N (%)**	**1–5**	19 (38.8)
	**6–10**	17 (34.7)
	**≥11**	13 (26.5)
**Full-time employees, N (%)**	**≤6**	6 (12.2)
	**7–10**	17 (34.7)
	**11–17**	10 (20.4)
	**≥18**	22 (44.9)
**Operation pattern, N (%)**	**Public construction and operation**	8 (16.3)
	**Public construction and private operation**	19 (38.8)
	**Private construction and operation**	22 (44.9)
**Types of service content, N (%)**	**≤7**	24 (49.0)
	**> 7**	25 (51.0)
**Types of service objects, N (%)**	**1–4**	27 (55.1)
	**5**	22 (44.9)
**Service object, N (%)**	**Self-care**	
	No	1 (2.0)
	Yes	48 (98.0)
	**Half-care**	
	No	3 (6.1)
	Yes	46 (93.9)
	**Total care**	
	No	8 (16.3)
	Yes	41 (83.7)
	**Particular care**	
	No	15 (30.6)
	Yes	34 (69.4)
	**Special care**	
	No	26 (53.1)
	Yes	23 (46.9)
**Number of elderly people served, N (%)**	**≤65**	25 (51.0)
	**> 65**	24 (49.0)
**Number of disabled elderly, N (%)**	**≤25**	21 (42.9)
	**> 25**	28 (57.1)
**Number of fund sources, N (%)**	**< 2**	10 (20.4)
	**≥2**	39 (79.6)
**Types of fund sources, N (%)**	**Service charges**	
	No	9 (18.4)
	Yes	40 (81.6)
	**Financial subsidies**	
	No	4 (8.2)
	Yes	45 (91.8)
	**Medical insurance funds**	
	No	29 (59.2)
	Yes	20 (40.8)
	**Social donations**	
	No	46 (93.9)
	Yes	3 (6.1)
	**Others**	
	No	46 (93.9)
	Yes	3 (6.1)
**Chain organization**	**No**	25 (51.0)
	**Yes**	24 (49.0)

Table 2 summarized the results of social capital scores of 49 organizations. Most organizations had a high score in norm (81.6%), participation (61.2%), trust (65.3%), common language (65.3%), and social capital (63.3%). The organizations with high scores in support and common vision dimensions were 49.0 and 55.1%, respectively.

**Table 2:** Social capital scores of 49 organizations

Variables	Scores
**Social capital (0–125), N (%)**	114.06 ± 9.82
Hight	31 (63.3)
Low	18 (36.7)
**Participation (0–30), N (%)**	25.86 ± 4.64
Hight	30 (61.2)
Low	19 (38.8)
**Trust (0–25), N (%)**	23.55 ± 2.42
Hight	32 (65.3)
Low	17 (34.7)
**Support (0–20), N (%)**	17.16 ± 2.52
Hight	24 (49.0)
Low	25 (51.0)
**Norm (0–15), N (%)**	14.71 ± 0.68
Hight	40 (81.6)
Low	9 (18.4)
**Common language (0–20), N (%)**	18.71 ± 1.86
Hight	32 (65.3)
Low	17 (34.7)
**Common vision (0–15), N (%)**	14.06 ± 1.56
Hight	27 (55.1)
Low	22 (44.9)

### Results of spearman correlation analysis

As shown in Table 3, spearman correlation analysis revealed that social capital was negatively correlated with region, establishment time, service content types, the number of elderly people served, and chain organization. However, only establishment time was significant in terms of social capital. Social capital was positively correlated with full-time employees, operation pattern, number of object categories, number of disabled elderly, and number of fund sources. We also conducted a correlation analysis for six dimensions of social capital, and the specific results were displayed in the supplementary file (2).

**Table 3:** The results of Spearman correlation analysis

Variable	Region	Establishment time	Full-time employees	Operation pattern	Types of service content	Types of service objects	Number of elderly people served	Number of disabled elderly	Number of fund sources	Chain organization	Social capital
**Region**	1.00	0.25	−0.24	−0.38^**^	0.06	−0.21	−0.23	−0.10	−0.30^*^	0.03	−0.21
**Establishment time**	0.25	1.00	−0.42^**^	− 0.12	0.26	− 0.17	− 0.21	− 0.24	− 0.08	−0.05	− 0.29^*^
**Full-time employees**	− 0.24	− 0.42^**^	1.00	0.30^*^	−0.26	0.45^**^	0.53^**^	0.43^**^	0.17	0.12	0.25
**Operation pattern**	−0.38^**^	− 0.12	0.30^*^	1.00	−0.06	0.44^**^	0.18	0.34^*^	0.20	−0.22	0.42^**^
**Types of service content**	0.06	0.26	−0.26	−0.06	1.00	−0.02	0.06	−0.02	− 0.29^*^	−0.10	− 0.21
**Types of service objects**	−0.21	− 0.17	0.45^**^	0.44^**^	− 0.02	1.00	0.26	0.45^**^	0.05	−0.06	0.34^*^
**Number of elderly people served**	−0.23	− 0.21	0.53^**^	0.18	0.06	0.26	1.00	0.52^**^	−0.01	0.18	−0.07
**Number of disabled elderly**	−0.10	− 0.24	0.43^**^	0.34^*^	−0.02	0.45^**^	0.52^**^	1.00	−0.03	− 0.06	0.35^*^
**Number of fund sources**	−0.30^*^	− 0.08	0.17	0.20	−0.29^*^	0.05	− 0.01	− 0.03	1.00	0.09	0.03
**Chain organization**	0.03	−0.05	0.12	−0.22	− 0.10	− 0.06	0.18	− 0.06	0.09	1.00	−0.12
**Social capital**	−0.21	− 0.29^*^	0.25	0.42^**^	−0.21	0.34^*^	−0.07	0.35^*^	0.03	− 0.12	1.00

* *p* < 0.05, ** *p* < 0.01

### Results of binary logistic regression model

In the likelihood ratio test results, the model was meaningful (*P* < 0.05) and the goodness of fit of the model is good (χ^2^ = 3.96, *P* > 0.05). Table 4 presented the findings of a binary regression logistic analysis of the link between social capital and potential influencing factors. In the univariate model, the logistic regression analysis indicated that integrated eldercare services with medical care SOs which established for more than 11 years (OR = 0.21; 95% CI: 0.05–0.94) were likely to report lower scores in social capital. SOs which were privately constructed and operated (OR = 3.57, 95% CI = 1.55–67.21) were more likely to report high scores in social capital. However, region, full-time employees, types of service content, types of service objects, number of elderly people served, number of disabled elderly, number of fund sources, chain organization were not statistically significant.

**Table 4:** Results of binary logistic regression model

	Crude OR (95% CI)	Adjusted OR (95% CI)
**Region**		
Urban	Ref.	Ref.
Rural	0.31 (0.08–1.14)	0.16 (0.02–1.49)
**Establishment time, years**		
1–5	Ref.	Ref.
6–10	2.15 (0.44–10.44)	5.95 (0.33–108.77)
≥ 11	0.21 (0.05–0.94)*	0.57 (0.05–6.83)
**Full-time employees**		
≤ 10	Ref.	Ref.
11–17	5.71 (0.92–35.48)	26.17 (0.76–904.54)
≥ 18	3.81 (0.99–14.65)	9.94 (0.37–270.00)
**Operation pattern**		
Public construction and operation	Ref.	Ref.
Public construction and private operation	5.14 (0.81–32.77)	2.77 (0.17–45.24)
Private construction and operation	3.57 (1.55–67.21)*	3.41 (0.19–61.15)
**Types of service content**		
≤ 7	Ref.	Ref.
> 7	0.75 (0.23–2.41)	0.50 (0.04–5.59)
**Types of service objects**		
1–4	Ref.	Ref.
5	3.16 (0.90–11.03)	0.88 (0.09–8.30)
**Number of elderly people served**		
≤ 65	Ref.	Ref.
> 65	0.66 (0.21–2.12)	0.01 (0.00–0.64)*
**Number of disabled elderly**		
≤ 25	Ref.	Ref.
> 25	3.3 (0.98–11.07)	14.01 (0.60–326.01)
**Number of fund sources**		
< 2	Ref.	Ref.
≥ 2	1.19 (0.29–4.95)	0.75 (0.06–9.97)
**Chain organization**		
No	Ref.	Ref.
Yes	1.91 (0.58–6.23)	1.12 (0.18–7.08)

* *p* < 0.05; *Ref.* Reference

After adjusting for all covariate variables, integrated eldercare services with medical care SOs which served more than 65 elderly people were likely to report lower score in social capital. There were no significant differences in region, establishment time, full-time employees, operation pattern, types of service content, types of service objects, number of disabled elderly, number of fund sources, chain organization.

## Discussion

This study aimed to analyze the correlates of external social capital in SOs providing integrated eldercare services with medical care in China. External social capital was found to be linked to integrated eldercare services with medical care SOs. Specifically, SOs served more than 65 elderly people would have more social capital.

This study found that there was no significant difference between rural and urban areas in social capital scores, which was different from a prior study indicating that rural areas had less accessible social capital [33]. The possible reason is that the rural economy is becoming more and more developed, and the economic gap between rural and urban areas is becoming smaller [33]. The finding indicated that equal attention should be paid to the integrated eldercare services with medical care SOs in rural and urban areas.

Additionally, we found that SOs which established for more than 11 years was not statistically significant after adjusting for all covariate variables. Unlike our findings, previous study found that establishment for longer time social organizations had more mature management levels [34]. A possible reason could be that that social capital was defined and quantified differently [35]. Our study defined external social capital as actual and potential resources that an organization obtains from a cooperative network established with other external stockholders and measured external social capital with six dimensions: participation, trust, support, norm, common language, and common vision. While, another study defined social capital as the average value of the extent to which individuals trust each other and participate in groups [34].

Although there were no significant differences between full-time employees and social capital. This was consistent with previous studies [26, 27], which revealed no significant differences between employees and external social capital. The possible reason was that as their scale expanded and their employment base grows, they become more involved in government activities and other social organizations as a part of their development [36]. Following that, more social capital was obtained.

The results indicated that there was no statistical significance between the three operation patterns. This was consistent with other results, which indicated that there were no significant difference in the total score of external social capital [26]. A possible explanation could be that the government has recently vigorously advocated different types of SOs to give full play to their advantages, emphasizing their benefits for healthy aging participation.

One of our findings was that there was no statistical significance between the type and quantity of service content and social capital. A study found that integrated eldercare services with medical care institutions pay attention to the diversified development of medical care and pension services [37]. Surprisingly, different from our findings, a study in the U. S. found that Pension institutions with many types of services have a higher level of social capital [38]. One possible explanation for this discrepancy was that whether the quantity and quality of services provided by elderly care institutions could meet the demands of elderly was not only critical to the institution’s social capital but also a significant factor in the elderly’s selection.

We also found that there was no statistical correlation between the types of service objects and social capital. Earlier research [5, 39, 40] had discovered that social organizations with different service objects had different relationship networks and different understanding of each other’s work status, which was different from our findings. The reason for this phenomenon may be the different classification of service objects. Previous studies divided service objects into normal or disabled [5, 39, 40], while our study divided service objects into five types.

SOs with more than 65 elderly people were more likely to reduce social capital scores. Meanwhile, SOs with more than 25 disabled people were more likely to increase social capital scores. Additionally, a study from Southeastern Brazil [41] found that disabled people had a lower level of social capital than the elderly. Additionally, our findings implied that we should pay more attention to disabled people from integrated eldercare services with medical care SOs.

According to our analyses, there is no statistically significant difference between the types of capital sources and social capital. Our study indicated that most SOs had service charges and financial subsidies. Some researchers believed that service charge was not conducive to long-term development and a stable and sustainable supply of pension services [42, 43]. This may be due to different social networks of organizations established at varying times and in different financing ways, as well as differences in their ability to provide medical and pension services [44]. Additionally, the government should pay more attention and strengthen its supervision [18, 45].

Compared with non-chain SOs, there was no statistical significance between chain SOs and social capital scores. A study demonstrated that the nursing quality and communication level of employees in chain elderly care institutions were relatively low, as was their social capital level [46]. Another study from California also pointed out that chain pension institutions had low social capital because of the lack of quality supervision [47].

In general, if the integrated eldercare services with medical care SOs had a large number of elderly people served, the level of external social capital was likewise high. Particular attention should be devoted to social organizations with short establishment time, few full-time employees, and single financing channels. Numerous factors influenced the social capital level of an organization. Moreover, the higher the social capital, the better the service quality provided by organization [45, 48].

Our study had several strengths. First, we used a comprehensive and effective scale to measure the social capital status integrated eldercare services with medical care SOs, including the basic situation of social organizations and social capital measurement, which might reduce the bias caused by the complexity of the project [13]. Additionally, to our knowledge, this was the first study to explore the social capital of integrated eldercare services with medical care SOs, which stimulated SOs to play a huge role in elderly care services, cultivated and enriched the external social capital stock of SOs, and provided more insights into population aging.

However, there were a few limitations that should be acknowledged. First, this study selected 1–3 core members familiar with the organization to finish in the questionnaire. Due to limited survey funds, time and, coordination degree of respondents, only one core member of some organizations completed the questionnaire, rendering in a small number of samples and the survey results unilateral. Future studies should include as many core members as feasible to ensure the survey’s credibility and authenticity. Second, because this study was conducted only in Anhui province, cautions were required when generalizing our results to other regions or countries.

## Conclusion

The medical and nursing pension social organizations in Anhui Province were mostly established for 1–5 years. Most numbers of full-time employees in organizations were more than 16. The objects of organizations were mainly self-care, half-care, and total care, and their primary funding sources were mainly financial subsidies and service charges. Our study also found that we should consider the impact of types of service objects on social capital and its dimensions. We recommend future studies to focus on mechanisms for enriching financing channels, diversifying the content of pension services, improving the construction of talent teams, and actively establishing pension social organizations and industry associations. Our study may assist in developing strategies to provide suggestions and references for external social capital cultivation.

## Supplementary Information

**Additional file 1.**

**Additional file 2.**

## Data Availability

All data generated or analysed during this study are included in this published article [and its supplementary information files].

## Abbreviations

SOs: Social organizations
95% CI: 95% confidence interval
Ref.: Reference
OR: Odds ratio
