# Supplementary material for: Correlations of external social capital in social organizations providing integrated eldercare services with medical care in China
Source: BMC Health Serv Res. 2022 Jan 25;22:101. doi: 10.1186/s12913-022-07508-2 (PMC8787872; doi:10.1186/s12913-022-07508-2)
Supplement: Supplementary file 1 — Additional file 1. [file 12913_2022_7508_MOESM1_ESM.docx]

| **Questionnaire Number: AH□□□□□□□□□□ Investigator Number:** □□ |
| --- |
| **Survey area： Province City District（County）** |

**Questionnaire of social organizations providing integrated eldercare services with medical care**

**School of Health Services Management, Anhui Medical University**

**Questionnaire of social organizations providing integrated eldercare services with medical care**

Hello! We are investigators from the school of health management of Anhui Medical University. Now we are carrying out a questionnaire survey on social organizations (SOs) providing integrated eldercare services with medical care. This survey is anonymous. The survey results are not for any organization or individual, but only for academic research. There is no right or wrong in your answer. There is no need to worry about it in the filling process, just fill it out truthfully according to the actual situation. Thank you for your support and cooperation.

**Part A Basic information of the SOs**

| **A** **Basic information** |
| --- |
| **A1 Name of organization:** |
| **A2 Region：**①Urban；②Rural |
| **A3 Establishment time:** years month |
| **A4 The number of full-time employees：** |
| **A5 The operation pattern of organization:** |
| ①Public construction and operation ②Public construction and private operation  ③Private construction and operation |
| **A6 Service content: (Multiple choices are allowed)** |
| ①Life care ②Spiritual consolation ③Health management ④Medical care  ⑤Hospice service ⑥Social work ⑦Leisure and Entertainment ⑧Cultural education  ⑨Protection of rights and interests ⑩Others |
| **A7 Types of service objects: (Multiple choices are allowed)** |
| ①Self-care ②Half-care ③Total care ④Particular care ⑤Special care |
| **A8.1 Number of elderly people serve:**  **，**  **A8.2 Including the number of disabled elderly:** |
| **A9 Types of fund sources: (Multiple choices are allowed)** |
| ①Service charges ②Financial subsidies ③Medical insurance funds  ④Social donations ⑤Others |
| **A10 Is it a chain organization？** ①Yes ②No |

**Part B External social capital of SOs**

| **B1 Participation** | | | | | |
| --- | --- | --- | --- | --- | --- |
| **Entries:** | ①Strongly disagree | ②Somewhat disagree | ③Undecided | ④Somewhat agree | ⑤Strongly agree |
| **B1.1** The organization regularly participates in activities organized by the health commission. |  |  |  |  |  |
| **B1.2** The organization regularly participates in activities organized by the medical security bureau. |  |  |  |  |  |
| **B1.3** The organization regularly participates in activities organized by street or community neighborhood committees. |  |  |  |  |  |
| **B1.4** The organization regularly organizes activities organized by other government departments. |  |  |  |  |  |
| **B1.5** The organization regularly participates in activities organized by the Federation of social organizations. |  |  |  |  |  |
| **B1.6** The organization often participates in experience exchange activities among social organizations. |  |  |  |  |  |
| **B2 Trust** | | | | | |
| **Entries:** | ①Strongly disagree | ②Somewhat disagree | ③Undecided | ④Somewhat agree | ⑤Strongly agree |
| **B2.1** The organization believes in the working ability of other member units of the health commission. |  |  |  |  |  |
| **B2.2** The organization believes in the working capacity of the medical security bureau. |  |  |  |  |  |
| **B2.3** The organization believes in the professional ability of street or community neighborhood committees to carry out the cause of aging. |  |  |  |  |  |
| **B2.4** The organization believes in the capacity of other government departments. |  |  |  |  |  |
| **B2.5** The organization believes in the working capacity of the Federation of social organizations. |  |  |  |  |  |
| **B3 Support** | | | | | |
| **Entries:** | ①Strongly disagree | ②Somewhat disagree | ③Undecided | ④Somewhat agree | ⑤Strongly agree |
| **B3.1** The organization has received resources provided by relevant government departments (such as funds, places, information, technology, etc.). |  |  |  |  |  |
| **B3.2** Other social organizations have provided financial support to the organization. |  |  |  |  |  |
| **B3.3** Other social organizations have provided information support to the organization. |  |  |  |  |  |
| **B3.4**Other social organizations have provided technical support to the organization. |  |  |  |  |  |
| **B4 Norm** | | | | | |
| **Entries:** | ①Strongly disagree | ②Somewhat disagree | ③Undecided | ④Somewhat agree | ⑤Strongly agree |
| **B4.1** The superior department shall supervise the work of the organization according to the needs. |  |  |  |  |  |
| **B4.2** The superior department will praise the organization according to the supervision results. |  |  |  |  |  |
| **B4.3** The superior department will rectify the organization according to the supervision results. |  |  |  |  |  |
| **B5 Common language** | | | | | |
| **Entries:** | ①Strongly disagree | ②Somewhat disagree | ③Undecided | ④Somewhat agree | ⑤Strongly agree |
| **B5.1** There are common professional symbols and terms among social organizations in this field. |  |  |  |  |  |
| **B5.2** The organization understands the work status of other social organizations in the field of integrated elderly care and medical care. |  |  |  |  |  |
| **B5.3** The organization agrees with the service achievements of other social organizations providing integrated elderly care and medical care. |  |  |  |  |  |
| **B5.4** The organization agrees with the strategic plan for elderly care services formulated by civil affairs and other superior departments. |  |  |  |  |  |
| **B6 Common Vision** | | | | | |
| **Entries:** | ①Strongly disagree | ②Somewhat disagree | ③Undecided | ④Somewhat agree | ⑤Strongly agree |
| **B6.1** The work carried out by the organization is often recognized by relevant units in the street or community. |  |  |  |  |  |
| **B6.2** For the key issues of elderly care services, the superior department will solicit the opinions of the organization when making decisions. |  |  |  |  |  |
| **B6.3** The organization and other organizations can reach an agreement on the government's decision-making related to the cause of aging. |  |  |  |  |  |

调查员：

质控员：

调查时间：
