# Supplementary material for: Correlations of external social capital in social organizations providing integrated eldercare services with medical care in China
Source: BMC Health Serv Res. 2022 Jan 25;22:101. doi: 10.1186/s12913-022-07508-2 (PMC8787872; doi:10.1186/s12913-022-07508-2)
Supplement: Supplementary file 2 — Additional file 2. [file 12913_2022_7508_MOESM2_ESM.docx]

**Table S1. The all results of Pearson correlation analysis**

| **Variabled** | **Region** | **Establishment time** | **Full-time employees** | **Operation pattern** | **Types of service content** | **Number of categories of object** | **Number of elderly people served** | **Number of disabled elderly** | **Number of fund sources** | **Chain organization** | **Participation** | **trust** | **support** | **norm** | **Common language** | **Common xision** | **Social captia** | |
| --- | --- | --- | --- | --- | --- | --- | --- | --- | --- | --- | --- | --- | --- | --- | --- | --- | --- | --- |
| **Region** | 1.00 | 0.25 | -0.24 | -0.38^**^ | 0.06 | -0.21 | -0.23 | -0.10 | -0.30^*^ | 0.03 | -0.25 | 0.01 | -0.13 | -0.09 | -0.21 | -0.05 | -0.21 | |
| **Establishment time** | 0.25 | 1.00 | -0.42^**^ | -0.12 | 0.26 | -0.17 | -0.21 | -0.24 | -0.08 | -0.05 | -0.41^**^ | 0.01 | -0.05 | 0.01 | -0.29^*^ | -0.21 | -0.29^*^ | |
| **Full-time employees** | -0.24 | -0.42^**^ | 1.00 | 0.30^*^ | -0.26 | 0.45^**^ | 0.53^**^ | 0.43^**^ | 0.17 | 0.12 | 0.22 | 0.06 | 0.19 | 0.22 | 0.17 | 0.20 | 0.25 | |
| **Operation pattern** | -0.38^**^ | -0.12 | 0.30^*^ | 1.00 | -0.06 | 0.44^**^ | 0.18 | 0.34^*^ | 0.20 | -0.22 | 0.42^**^ | 0.11 | 0.30^*^ | 0.25 | 0.35^*^ | 0.20 | 0.42^**^ | |
| **Types of service content** | 0.06 | 0.26 | -0.26 | -0.06 | 1.00 | -0.02 | 0.06 | -0.02 | -0.29^*^ | -0.10 | -0.15 | -0.10 | -0.26 | -0.05 | -0.11 | -0.15 | -0.21 | |
| **Number of categories of object** | -0.21 | -0.17 | 0.45^**^ | 0.44^**^ | -0.02 | 1.00 | 0.26 | 0.45^**^ | 0.05 | -0.06 | 0.31^*^ | 0.12 | 0.15 | 0.26 | 0.39^**^ | 0.20 | 0.34^*^ | |
| **Number of elderly people served** | -0.23 | -0.21 | 0.53^**^ | 0.18 | 0.06 | 0.26 | 1.00 | 0.52^**^ | -0.01 | 0.18 | -0.03 | -0.14 | -0.10 | 0.24 | -0.05 | -0.04 | -0.07 | |
| **Number of disabled elderly** | -0.10 | -0.24 | 0.43^**^ | 0.34^*^ | -0.02 | 0.45^**^ | 0.52^**^ | 1.00 | -0.03 | -0.06 | 0.32^*^ | 0.13 | 0.27 | 0.31^*^ | 0.25 | 0.17 | 0.35^*^ | |
| **Number of fund sources** | -0.30^*^ | -0.08 | 0.17 | 0.20 | -0.29^*^ | 0.05 | -0.01 | -0.03 | 1.00 | 0.09 | -0.20 | -0.05 | 0.24 | 0.16 | 0.20 | 0.22 | 0.03 | |
| **Chain organization** | 0.03 | -0.05 | 0.12 | -0.22 | -0.10 | -0.06 | 0.18 | -0.06 | 0.09 | 1.00 | -0.11 | -0.21 | -0.03 | 0.17 | -0.18 | 0.07 | -0.12 | |
| **Participation** | -0.25 | -0.41^**^ | 0.22 | 0.42^**^ | -0.15 | 0.31^*^ | -0.03 | 0.32^*^ | -0.20 | -0.11 | 1.00 | 0.36^*^ | 0.48^**^ | 0.20 | 0.44^**^ | 0.26 | 0.82^**^ | |
| **Trust** | 0.01 | 0.01 | 0.06 | 0.11 | -0.10 | 0.12 | -0.14 | 0.13 | -0.05 | -0.21 | 0.36^*^ | 1.00 | 0.37^**^ | 0.17 | 0.26 | 0.42^**^ | 0.64^**^ | |
| **Support** | -0.13 | -0.05 | 0.19 | 0.30^*^ | -0.26 | 0.15 | -0.10 | 0.27 | 0.24 | -0.03 | 0.48^**^ | 0.37^**^ | 1.00 | 0.15 | 0.44^**^ | 0.40^**^ | 0.73^**^ | |
| **Norm** | -0.09 | 0.01 | 0.22 | 0.25 | -0.05 | 0.26 | 0.24 | 0.31^*^ | 0.16 | 0.17 | 0.20 | 0.17 | 0.15 | 1.00 | 0.35^*^ | 0.41^**^ | 0.38^**^ | |
| **Common language** | -0.21 | -0.29^*^ | 0.17 | 0.35^*^ | -0.11 | 0.39^**^ | -0.05 | 0.25 | 0.20 | -0.18 | 0.44^**^ | 0.26 | 0.44^**^ | 0.35^*^ | 1.00 | 0.77^**^ | 0.72^**^ | |
| **Common vision** | -0.05 | -0.21 | 0.20 | 0.20 | -0.15 | 0.20 | -0.04 | 0.17 | 0.22 | 0.07 | 0.26 | 0.42^**^ | 0.40^**^ | 0.41^**^ | 0.77^**^ | 1.00 | 0.66^**^ | |
| **Social captia** | -0.21 | -0.29^*^ | 0.25 | 0.42^**^ | -0.21 | 0.34^*^ | -0.07 | 0.35^*^ | 0.03 | -0.12 | 0.82^**^ | 0.64^**^ | 0.73^**^ | 0.38^**^ | 0.72^**^ | 0.66^**^ | 1.00 | |
| * p<0.05, ** p<0.01 | | | | | | | | | | | | | | | | | |  |
